# Multiple Myeloma Exemplifies a Model of Cancer Based on Tissue Disruption as the Initiator Event

**DOI:** 10.3389/fonc.2018.00355

**Published:** 2018-09-10

**Authors:** Jean-Pascal Capp, Régis Bataille

**Affiliations:** ^1^LISBP, UMR CNRS 5504, UMR INRA 792, INSA Toulouse, University of Toulouse, Toulouse, France; ^2^Faculty of Medecine, University of Angers, Angers, France

**Keywords:** multiple myeloma, MGUS, oncogenesis, plasma cells, endosteal niche, bone lesion, heterogeneity, gene expression noise

## Abstract

The standard model of multiple myeloma (MM) oncogenesis is based on the genetic instability of MM cells and presents its evolution as the emergence of clones with more and more aggressive genotypes, giving them surviving and proliferating advantage. The micro-environment has a passive role. In contrast, many works have shown that the progression of MM is also characterized by the selection of clones with extended phenotypes able to destroy bone trabeculae, suggesting a major role for early micro-environmental disruption. We present a model of MM oncogenesis in which genetic instability is the consequence of the disruption of normal interactions between plasma cells and their environment, the bone remodeling compartment. These interactions, which normally ensure the stability of the genotypes and phenotypes of normal plasma cells could be disrupted by many factors as soon as the early steps of the disease (MGUS, pre-MGUS states). Therapeutical implications of the model are presented.

## Introduction

Cancer is increasingly viewed as a tissue disease. This view results from numerous studies where tissue disruption can be either the inducer or the repressor of the cancerous state (1). Nevertheless, the genetic origin of the disease remains widely accepted (the necessary pre-existence of mutated cells and the initiator role of mutations are most of the time not called into question) in spite of new data showing (a) the presence of oncogenic mutations in normal tissues (2–4) and the ability of these tissues to eliminate mutant cells to prevent tumor initiation (5), (b) the development of pre-cancerous lesions without oncogenic mutations (6), or (c) the presence of epigenetic (7), gene expression (8) or micro-environmental (9) alterations that might precede the emergence of genetically abnormal cells, that further question the genetic model of cancer initiation. Epigenetic alterations especially are increasingly acknowledged as being able to initiate transformation, as genetic alterations do, by providing the gene expression plasticity necessary to provide stochastic oncogenic epigenetic changes (10). Recent works on lung cancer showed that chronic cigarette smoke-induced epigenomic changes necessarily precede oncogene-induced transformation (11). *KRAS* mutations are inefficient to produce tumors without preexisting epigenetic alterations.

Several alternative models soon suggested considering epigenetic alterations (12) or tissue disruption (13, 14) as initiator events. The models based on tissue disruption are especially inspired by works showing that the sole tissue disruption is able to produce tumors in many experimental models, especially in leukemogenesis when the bone is affected (15–18). These models then differ when considering the role of genetic alterations: some acknowledge their crucial role in cancer progression without their necessary pre-existence (1, 14) while others consider them mainly as a side-effect of cancer development (13).

It has been recently proposed to add a new element in this complex interplay. Since a decade, stochastic gene expression (SGE) (gene expression noise) is recognized as a source of cellular heterogeneity and phenotypic plasticity that are hallmarks of both embryonic and adult stem cells (19). Variation in gene expression arising from transcriptional noise and network fluctuation and the associated phenotypic heterogeneity accounts for stochasticity of cell fate decisions in stem and progenitor cells (20). Moreover, the degree of SGE is modulated during development and differentiation: many studies now showed that following a phase of highly and widespread SGE, cells progressively transit toward a more homogeneous, coordinated and restricted gene expression patterns (21–23) associated with a more restrictive chromatin (24). Of note, when hematopoietic stem or progenitors cells are induced to differentiate, a transient state is characterized by an increased in SGE (22, 23). Highly variable expression seems necessary for the necessarily large developmental “choices” of stem cells and in contrast as differentiation progresses, expression patterns become more tightly constrained, well-defined and less diverse (25).

Cellular interactions have been recently showed as major determinants in constraining and decreasing SGE and possess all the requirements to be considered as the main “constraints” leading to stable differentiated states. Recent works in Drosophila suggest that cellular communications are essential in stabilizing and homogenizing gene expression during cell differentiation (26). A similar mechanism was already described in *Caenorhabditis elegans* embryos where strong signaling is essential to maintain low expression variability and to ensure reliable neuroblast development (27). Also, an initial phase of stochastic expression of individual genes preceding signal reinforcement through Fgf4 that segregates early lineages has also been demonstrated in mouse blastocysts (28). Direct cell contacts through gap junctions also spatially coordinate prolactin gene expression in pituitary adult tissue (29). Moreover, enzymatic digestion of extracellular proteins or pharmacological inhibition of gap junctions reduced transcriptional coordination between cells (29), showing that perturbation of cell communication can enhance SGE and phenotypic heterogeneity among differentiated cells. Overall a model of cancer where disruption of cellular interactions is the initiator event by producing phenotypic plasticity and less differentiated cancer cells (that could be named cancer stem cells defined as cells exhibiting increased epigenetic plasticity and increased SGE because they are no more controlled by the microenvironment) is entirely possible and has been previously proposed (1, 14, 30). Considering epigenetic or gene expression alterations as the initiator events (10) without considering their likely origins in the failure to maintain tissue homeostasis and the necessary accompanying tissue disruption misses integrating the full micro-environmental contribution to cancer initiation.

Multiple myeloma (MM) is one the most well-characterized cancers at the molecular, cellular and environmental levels (31). In contrast with other intensively studied cancers such breast cancers, a large body of works provides valuable sources to consider the possible role of the micro-environment in MM initiation. Moreover, the pre-malignant steps known as monoclonal gammopathy of undetermined significance (MGUS) or even pre-MGUS phases are now better characterized and allow reinterpreting the evolution of the disease together with many data at various steps obtained in recent years. Finally, the knowledge accumulated on the normal counterpart [normal plasma cells (PC) on their niche] and the animal models mimicking the human disease all motivate the choice of MM in this review. Its purpose is to present MM as a good illustration of a model of cancer based on tissue disruption as the initiator event.

## Hallmarks of MM

MM is a B/plasma cell malignancy characterized by the accumulation of malignant PC within the bone marrow. MM cells are mainly characterized by their aberrant genotype, morphotype (proliferation/differentiation status), phenotype and extended phenotype, when compared with their normal counterparts, the long-lived PC residing inside the bone marrow (31, 32). Indeed MM (i) presents with complex karyotypes compiling many chromosomal abnormalities including aneuploidy (trisomies/deletions), immunoglobulin heavy chain (IGH) translocations and gene mutations; (ii) retains the capacity for a slow residual proliferation, then achieving less differentiation (nuclear-cytoplasmic asynchrony morphotype); (iii) has lost normal PC antigens while expressing many lineage infidelities; (iv) progress at the expense of both normal PC and bone tissue. Indeed, evolution of MM is mainly characterized by the disappearance of normal PC inside their physiological niche, the hematopoietic/endosteal/osteoblastic niche, whereas bone trabeculae surrounding MM cells are irreversibly destroyed. The disappearance of both normal PC and bone trabeculae explains the most deleterious manifestations of overt MM, hypo-gammaglobulinemia and pure lytic bone lesions (LBL) and represents the hallmark of MM, designed by its extended phenotype (33).

### Genotype

As many cancers, MM is characterized by numerous genetic alterations (34). These include cytogenetically visible changes such as chromosome gains (mainly trisomy 5, 1q+), losses (mainly −13q, del16) and IGH translocations, and subtle changes in DNA sequence (Ras and p53 mutations for example). Some of these chromosome changes are observed at the early stages of the disease (MGUS) and are defined as primary; some others occur later (secondary changes).

This view is at the origin of the standard molecular model of MM (see below). MM appears to develop genetic instability mainly at the chromosomal level: the karyotype of the majority of the patients is aneuploid, aneuploidy being the readout of an underlying chromosomal instability. Taking such aneuploidy into consideration, at least three types of MM can be considered (i) non-hyperdiploid (mainly with translocations and deletions), with too much genetic instability; (ii) hyperdiploid with just right instability; and (iii) diploid (mainly translocations 11/14) with low genetic instability (35). Of note, this degrees of genetic instability correlates with the presenting features and clinical outcome of the patients: the non-hyperdiploid form presents as the most aggressive MM, hyperdiploid with the most LBL and diploid being close to normal, with long pre-clinical and clinical history. Gene mutations are frequent, including mainly *RAS* and *P53* mutations, and are also primary or secondary, increasing the genetic heterogeneity of the MM clone. Whereas, some of these point mutations are of prognostic value at diagnosis (*KRAS* mutations), some others occur later, mainly at the time of extra-medullary evolution of the disease.

### Morphotype

MM cells retain the capacity to slowly proliferate but have a survival advantage. Indeed, MM cells present a special proliferating and survival status different from that of their normal counterparts, the long-lived bone marrow PC and their precursors, normal plasmablasts (36). Normal plasmablasts, the PC progenitors, are highly proliferating cells [all cycling, with labeling index (LI) as high as 30%], differentiate into pro-PC (PC precursors), then mature into long-lived PC. These long-lived PC do not proliferate anymore (cycle exit and proliferation arrest). On the other hand, proliferation arrest is never observed in MM cells, MM cell are unable to exit the cell cycle as opposed to normal PC, thus they retain the potential for a low rate of proliferation (LI between 0.5 and 3%). This “residual” (rather than true increased) proliferation is not observed in MGUS cells (37). Of note, this residual proliferation is well-illustrated at the morphological level, by the nuclear-cytoplasmic asynchrony observed in MM cells but not in MGUS cells. Finally, this residual proliferation, simply evaluated by the LI, explains the long-term accumulation of MM cells and is of strong prognostic value at any stage of disease transitions, from MGUS to smoldering MM (SMM) to overt MM to relapse.

Since the pioneer work of BGM Durie, the Mayo Clinic's group has performed extensive studies of the LI in MGUS, SMM, and overt MM at any stage of the disease: diagnosis, early and late relapses with frequently extra-medullary locations (38). These studies have shown that an increase of the LI characterized each new phase of MM oncogenesis (as a three phases process) and that the LI was the most potent prognostic factor in MM at any stage of the disease. This group has completed its model by measuring both proliferation and apoptosis of PC of individuals with MGUS and patients with MM (39). This study has confirmed that the transition from MGUS to SMM to overt MM was associated to both an increase of the LI and a decrease of the apoptotic index. Overall, this group has developed a model of a MM cell growth index that relates both proliferation and apoptosis to disease activity.

Of note, not only the majority of MM cells retains the potential for a low rate of proliferation, proliferating advantage illustrated by the LI, but also a tiny fraction of the MM clone (< 4% of total cells) actively replicates, mainly in the close vicinity of bones. Although the capacity of this small fraction of MM cells to proliferate does not exceed that of normal plasmablasts (since this is already optimal, with LI close to 30%), it is sufficient to “feed” the low proliferating compartment. The survival advantage of MM cells is also illustrated by the overexpression of either Mcl1 or Bcl2, two major anti-apoptotic proteins, by MM cells (40).

### Phenotype

MM cells progressively achieve an aberrant and heterogeneous phenotype. Indeed, they progressively loss PC antigens (CD19, CD20, CD27), except CD138 which is over-expressed, while expressing epithelial, T and NK cell antigens, Cancer Testis Antigens (CTA), CD28 and CD56 (survival phenotype/ancillary pathways). Furthermore, they have an aberrant kinome-phosphatasome profile since they aberrantly express either CD221/IGF1R or CD117/c-Kit while progressively lacking the phosphatase CD45 (41).

The phenotype of MM cells has a strong impact on clinical outcome of MM patients. As soon as the diagnosis, the loss of PC antigens (CD27), lineage infidelity (expression of CTA or of the T cell antigen CD28), the aberrant expression of CD221/IGF1R and the lack of CD45 define aggressive MM with poor prognosis and short survival. With disease progression, these aggressive types of MM become dominant, whereas the less aggressive types of MM disappear, that is those retaining PC antigens like CD20, CD27, those presenting CD117/c-kit rather than CD221/IGF1R or those retaining CD45. Thus, aggressive phenotypes exist at diagnosis and are selected during the evolution of MM to become dominant (in agreement with the concept of tiding clones).

The unique phenotype of MM cells not only influences the clinical outcome of MM but influences also the clinical presentation of the disease, especially bone or extra-medullary involvement. Indeed, some phenotypic features make MM cells able to develop complex interactions with cells of the microenvironment, mainly stromal cells, macrophages, dendritic cells but also endothelial cells. This interactive capacity of MM cells extends their standard phenotype toward bone cells, either osteoclasts (OC) or osteoblasts (OB), giving MM cells a specific extended phenotype, that is the capacity to destroy bone trabeculae. Among these interactions, the expression of CD56/N-cadherin and the production or induction by MM cells of factors able to activate OC or to inhibit OB play a major role (see the section on bone environment).

In addition to genotypes, the different phenotypes of MM cells represent another major source of inter- and intra-tumoral heterogeneity. This heterogeneity impacts clinical presentation, drug sensitivity and clinical outcome of the patients. Of importance is the high level of intraclonal heterogeneity, especially well-documented in patients with high-risk MM. This intra-clonal phenotypic heterogeneity is most often seen for the expression of CD45, a phosphatase which regulates the effects of MM cell growth factors on MM cells, actually which regulates the availability of the major growth factors IL6 and IGF1 (as nutrients) for MM subclones. This regulation operates through the capacity of CD45 to facilitate either SRC kinase activity (IL6 signaling) when present at the surface of MM cells, or RTK activity (IGF1R/CD221 and IGF1 signaling) when absent since CD45 is a potent inhibitor of IGF1R (CD221) aberrantly expressed on MM cells (42, 43).

### “Extended” phenotype

During the last 20 years, a lot of works has been devoted to the mechanisms of MM bone disease, and major discoveries have been made. It has been shown that the capacity of MM cells to destroy bone trabeculae is not simply due to the stimulation of OC (and of bone resorption), as previously emphasized, but rather to the suppression of OB (and of new bone formation), thus to an uncoupled bone remodeling (44–46). Furthermore, this suppression of OB and of bone formation is specific to MM. Indeed it is not observed in other cancers and leads to pure LBL, not observed in bone metastasis. Another important discovery is that the mechanisms of OB suppression, as those of OC stimulation, although specific to MM, were not unique. They do not simply result from the direct effects of MM cells on bone cells through the release of deleterious “bone factors” (such as Wnt signaling inhibitors), but rather from the complex interactions between MM cells and their microenvironment, activated accessory cells surrounding MM cells, mainly stromal cells, able to release deleterious bone factors such as RankL (activating OC) and activin A (suppressing OB). Thus, beyond their aberrant genotype, morphotype and phenotype, MM cells are also characterized by a specific “extended phenotype,” which is represented at the tissue level by pure LBL, and at the cellular level by the specific suppression of OB. It is interesting to note that both specific cellular targets of the MM process, normal PC and bone cells, reside inside the bone remodeling complex (BRC), the hematopoietic/osteoblastic/endosteal niche. This includes OC, the OC/osteocytes/lining cells complex, rather than OB alone, stromal cells and immune cells. Obviously, the BRC appears as the tissue of reference specifically targeted by the MM process (47, 48).

#### Within hematological malignancies, LBL are the hallmark of MM

MM is the only hematological malignancy associated with LBL, and the mechanisms of bone destruction are well-documented both at the cellular and molecular levels. Almost all MM patients will develop LBL during the evolution of their disease. Bone trabeculae rather than cortical bones are the target of the MM process. Actually, MM patients present with more or less LBL (bone heterogeneity) in relation to the other characteristics of their tumors, especially genetic instability. For example, hyperdiploid MM are more osteolytic that those with 14q32 translocations. It is now well-established that a MM-induced uncoupling process, that is an increased bone resorption with a decreased bone formation, is at the origin of LBL. This uncoupling is only observed in the close vicinity of MM cells. The mechanisms of LBL have been extensively and recently reviewed (49).

MM cells activate OC directly or indirectly through the microenvironment. MM cells produce potent OC activating factors, such as MIP alpha, IL3, IL7… MM cells produce or induce RankL, the most potent activator of OC on stromal cells, through VLA4/VCAM1 interactions. The presence of RankL on stromal cells is the proof of the existence of a reactive stroma in MM, as it is observed in the majority of carcinomas (50). No direct contact between MM cells and OC is necessary since MM cells release MMP7 and heparanase able to solubilize RankL on stromal cells. MM cells also inhibit the production of OPG/osteoprotegerin, a decoy receptor, inhibitor of RankL.

#### MM cells suppress OB

MM cells aberrantly express CD56/Ncam, a potent inducer of apoptosis in OB through homotypic interactions since OB and those belonging to the “lining cell complex” express CD56 too. MM cells also aberrantly express N-Cadherin, an inhibitor of OB differentiation. MM cells release soluble inhibitors of OB differentiation (DKK1, FRZB…) or induce such inhibitors in stromal cells (activin A, Gsf1, sclerostin), another proof of the existence of a reactive stroma in MM. Several of these inhibitors inhibit the Wnt pathway, a pathway essential for the differentiation of OB. MM bone disease can be presented as a Wnt-associated disease.

The capacity of MM cells to directly or indirectly inhibit bone formation is specific of MM, although many carcinomas have the capacity to stimulate bone resorption, directly or indirectly, in a similar way to that of MM (51–53). MM induced LBL present as pure LBL. Unlike other cancers presenting with sclerotic or mixed bone metastasis, such as prostate and breast cancers, MM represents the only cancerous diseases with pure LBL. This indicates that changes that occur in mesenchymal stem cells and OB may be unique to MM and not shared with other cancers. For these reasons, MM bone disease appears as a specific one, suggesting that the mechanisms explaining OB suppression are probably very close to those explaining the pathogenesis of MM. The proximity between the mechanisms of MM bone disease and MM pathogenesis is supported by the results of the genomics studies comparing the gene expression profile of normal bone marrow PC, of MGUS PC and of MM cells. In these comparative studies, genes coding for inhibitors of OB differentiation (especially for FRZB proteins family) have the highest discriminant expression (x170 increased expression in MM cells compared to normal or MGUS PC), comparable to the aberrant CCND over-expression (× 130-fold increase) (54).

#### Few MM do not develop LBL, while true sclerotic MM remain exceptional

MM lacking LBL remains exceptional. Of note, quantitative histology shows that there is no suppression of OB differentiation in these MM lacking LBL. OB remain active (“osteoblastic” MM) (49). This is well-illustrated by the fact that these patients present increased osteocalcin levels, compared to lower levels observed in patients with LBL. These MM have a better prognosis than osteolytic MM, are always of the lambda subtype like sclerotic MM, suggesting that they belong to the same family. This is also supported by the association between the lack of LBL and some special polymorphisms. It is worthwhile to note that sclerotic myeloma frequently presents as solitary myeloma. Overall, these observations suggest that maintenance/stimulation of OB at least protects from aggressive MM or even blocks disease transition from MGUS/SMM to overt MM. This is in agreement with the observation that OB produce inhibitors of MM cell growth like Decorin (see below).

### Evolution from MGUS

It has been recently shown that all MM cases emerge from a pre-existing state termed MGUS (or SMM, according to the extent of bone marrow involvement and serum monoclonal Ig levels) (55). This major discovery confirms the previous hypothesis of Salmon and Seligman (56), presenting the emergence of MM as a “2 hits phenomenon”: emergence of MGUS from an unknown pre-MGUS phase, then emergence of MM from a MGUS phase, after a more or less long period of time. Furthermore, MGUS turned out to present with an aneuploid genotype similar to that of MM cells. Finally, several convincing studies have shown significant abnormalities of bone remodeling in MGUS, revealing that the BRC is also involved at this early step of the disease (57–59). Considering these data, MGUS, now obligatory, constant, cannot appear any more as a simple and inconstant pre-malignant state of MM. MGUS presents as an intermediate state/step of malignancy between full (overt) malignancy that is MM and the normal state. Considering this new point of view, the research about putative pre-MGUS states appears of major interest.

### Normal counterpart and the concept of pre-MGUS

A lot of works have been devoted to the generation of normal PC, long-living inside the bone marrow, inside the hematopoietic/endosteal/osteoblastic niche (60–63). These long-lived PC represent the normal counterparts of MM cells, MM cells as their normal counterparts having accumulated numerous somatic mutations. These resting PC result from the differentiation *in situ* (in their niche, in the close contact with bone cells) of immature circulating plasmablasts. Of note, plasmablasts result from the fast activation of circulating memory B cells, which turn out to be the true precursors of long-lived PC. Both circulating memory B cells and plasmablasts can reside inside the “PC niche.” In this context, MM can be viewed as the malignant transformation of the normal process of plasmacytopoïesis. The MM transformation involves the early stages of plasmacytopoïesis (memory B cells) since the memory B cells of MM patients share with MGUS and MM cells the same aneuploid genotype. Pre-MGUS states exist, in the context of polyclonal activation of B cells, as polyclonal expansions of plasmablasts, which are reactive plasmacytoses (36). In Gaucher disease or in auto-immune thyroid diseases, the sequence “polyclonal B cell activation (pre-MGUS) > MGUS > overt MM” has been documented, by-passing the concept of “two-hit phenomenon” based on the specific activation of B cells (56). Finally, the abnormalities of Toll-like receptors expression on MM cells could suggest abnormal response to bacterial infections in MM patients (64).

## The standard “molecular” model of MM evolution and its limitations

### The model

The pathogenesis of MM from its pre-existing state, MGUS, and of MGUS from normal B and PC (2-hit process) (56) has been recently and thoroughly re-evaluated. A standard model of molecular pathogenesis is now proposed. According to this model, the initiating/primary genetic abnormalities hyperdiploïdy (mainly trisomies) and/or 14q32 IGH chromosomal translocations (mainly with five chromosomal partners) are the main transforming events that target the B cells involved into the generation of memory B cells within germinal centers (cytogenetic abnormalities which have been observed in the memory B cells of patients with MM) (34). The origin of these primary transforming events is supposed to be the DNA instability accompanying the DNA breaks associated to switch, somatic mutations and antigen receptor re-edition occurring in normal B cells. Of major importance, these primary events (i) are observed in almost 100% of precursor MM cells; (ii) are associated to overexpression of Cyclin D (1, 2, or 3) and Myc in precursor MM cells; (iii) are stable with time and (iv) are correlated with some presenting features and clinical outcome, making MM as many and multiple (they are different species of MM, see below). Subsequently, different “tiding” clones acquire new (secondary) genetic abnormalities according to a branching evolution model, in which only those with a better proliferation rate and survival advantage (the fittest) will invade. These secondary mutations, which occur during the transition from MGUS to overt MM, target plasmablasts, are associated with increased MYC expression, with sometimes activating mutations of RAS, BRAF or with chromosome 13q deletion. Further progression of MM is associated with other genetic (p53 point mutations) and/or epigenetic events (involving the methylation, histone acetylation process) (65, 66). These subsequent events increase the genomic instability of MM cells, their proliferation rate, their capacity to survive and decrease their dependence on the environment (bone marrow disruption, with extra-medullary evolution). In this point of view, de-differentiation, increase of proliferation and independence of the environment are considered as the consequences of increased genomic instability. Of note, as previously emphasized, primary cytogenetics (hyperdiploïdy, IGH translocations), although occurring at very early steps of the disease, impacts (i) the delay of transition from MGUS/SMM to overt MM and (ii) the presenting features (bone involvement in particular) and clinical outcome (survival) of patients. Thus, the early primary events delineate different subsets of MM in which secondary events will occur to influence particular evolution. In this context, MM presenting with t(11/14) are of particular interest because frequently diploids, with a sub-normal (mature) morphotype and phenotype and a long pre-clinical and clinical history, from MGUS to overt MM including the pre-switched IgM MM to PC leukemia.

### Limitations of the model

The standard model of MM evolution is presented as a “so-called” Darwinian branching model of tumor evolution, as previously proposed in carcinomas (65, 67). In such model, the evolution of MM is presented as the consequence of the invasion of the most aggressive clones from genetically unstable and “tiding” clones, aggressiveness impacting proliferation rate and dependence on the environment. Despite its major interest as the first and now standard model of MM pathogenesis, it presents with some limitations, especially to be a model not considering all the data currently available, because of its lack of consideration of the active role of the micro-environment.

In the MM standard model: (i) intrinsic genetic instability has the major role, generating genetic variations and (ii) within tiding clones, competition favors the fittest clone (survival advantage) thank to advantageous genetic variations. In this model, environment is destroyed as a consequence of MM progression, thus has also a passive role and no role as a “*primum movens*” of the process of evolution. Thus, the standard model is a model where genetics comes at first and where intrinsic genetic instability is the unique driving force, excluding any active role of the micro-environment.

Another important limitation of the standard model of MM evolution is to neglect the phenotypic heterogeneity of the MM clone (as marked as its genetic heterogeneity) and that of its extended phenotype. Indeed, evolution of MM is characterized by the emergence of clones with more aggressive phenotypes and extended phenotypes (high capacity to destroy bone trabeculae). The phenotypic initial and subsequent heterogeneity of the MM clone, especially CD45 heterogeneity which is the most important one, demonstrates the existence of a competition between subclones for limited resources (that are mainly nutrients/growth factors like IL6 and IGF1 whose effects on MM cells are discriminated by the presence or absence of CD45 on MM cells) from the microenvironment (exerting selection pressures), then selection of the most aggressive clones (68, 69, 70). Thus, the phenotype (especially the most heterogeneous one, that is the kinome-phosphatasome profile/CD45) of MM cells and their extended phenotype appear as critical elements of MM evolution. The most aggressive MM subclones, which will be selected during the evolution of MM, will be characterized by a strong capacity to destroy bone trabeculae and by their potential to grow thank to the best kinome-phosphatasome profile that is the lack of CD45 parallel to the aberrant expression of CD221/IGF1R (71).

The fact that the rate of proliferation and survival differs between the mass of MM cells and the tiny fraction of replicating MM cells within the MM clone suggests that this cell mass emerges from the tiny fraction of replicating MM cells through a process of natural selection. CD45, overexpressed on replicating MM cells, is the best candidate to be the target of selection. What is the origin of the selecting pressures exerted on replicating MM cells? Considering their well-documented importance in the biology of MM, bone and stromal environments represent good candidates to exert selecting pressures on replicating MM cells. Replicating MM cells are CD45+ and replicate in the close vicinity of bone and stromal cells. The role of stromal cells turns out to be a major one. Stromal cells (i) release Gal1 able to expand CD45– MM cells at the expense of CD45+ MM cells; (ii) express and release RankL, a potent activator of OC, and (iii) release activin A, a contraIL6 and inhibitor of OB, contraI IGF1R by themselves (stromal activin A twice in favor of IGF1 at the expense of IL6). In association with stromal cells, the MM ecosystem includes important immunological cells, such as macrophages/dendritic cells and T cells, cells which could influence the survival and proliferation of MM cells directly or through the bone micro-environment and through critical factors such as RankL (72–74).

There is now evidence of a vicious circle between bone and MM cells, bone environment accelerates MM cell growth and the selection of aggressive extended phenotypes. Bone cells (in the BRC) regulate MM cell growth since (i) OC support MM cell survival and growth and (ii) OB inhibit MM cell growth, through Decorin, a soluble pan-RTKs inhibitor, mainly a contra-IGF1R/CD221. Furthermore, there is also evidence that an excessive bone resorption in the endosteal niche accelerates MM “take” and selects more aggressive clones which destroy the bones (see below). Thus, bone barriers are attractive, supportive and selective of MM cells and this new concept by-pass the ancillary concept of “seed and soil”. All these studies represent the proof of bi-directional interactions between MM cells and the nearby bone cells that are permissive for tumor initiation and progression, establishing a positive feed-back loop that may be self-amplifying (vicious circle). Of note, this process occurs at the early stages of the disease since bone remodeling is abnormal as soon as the stage of MGUS.

In our point of view, a more complete explanation (model) of MM pathogenesis from obligatory MGUS has to reconcile the evolution (natural history) of MM not only with the molecular evolution of the MM clones (due to their genetic instability) during disease progression but also with the cellular and environmental changes/variations (especially in the BRC) characterizing MM progression. Second, it becomes also clear that the model has to question about the direct involvement of this microenvironment into the emergence of MM through the direct influence of this microenvironment on the genetic stability of MM cells. Finally, the model has to question about the mechanisms of emergence of MGUS from pre-MGUS states. The specific microenvironment of MM, the BRC, has to be included in the model. Overall, the model has to question about the putative role of the interactions (or lack of interactions) of MM cells into the appearance of their genomic instability, phenotypic heterogeneity and aggressive extended phenotypes, and that as a consequence of a process of tissue disruption. In summary, a more complete model has to include not only the genomic instability which generates genetic variations but also the different processes of selection of the fittest clones with the most adjusted phenotype through the micro-environment, and finally the role of the micro-environment on the occurrence of genetic instability and differentiation problems themselves.

## A new model in which tissue disruption is the initiator event

The BRC has a crucial importance in PC maturation through OC and their quiescence through OB (75, 76). Its normal functioning is necessary to PC differentiation and stabilization. Thus, is the slow accumulation of PC observed during the transition from MGUS to MM favored by the disruption of the interactions between bone cells (OC/OB) and PC which are physiologically important to maintain PC into a non-proliferating, differentiated state?

Moreover this equilibrium of the BRC is already severely disrupted in MGUS. Knowing the role of the interactions between PC progenitors and bone cells during plasmacytopoiesis, one can ask if early disruption of these interactions could be at the origin of PC differentiation and proliferation defects in MGUS. If this micro-environment is modified by an infection or a mechanical dysfunction, the equilibrium leading to well-matured and quiescent PC is disrupted and this process could generate non-fully differentiated PC. Moreover, other factors support the role of such disruption at the origin of MGUS. Indeed, it is well-documented that the incidence of MGUS increases with age, in relation to age-related decrease of the immune system efficacy. This parallels a decrease of bone mineral content, also in relation to an age-related excess of bone remodeling.

Finally it is also necessary to question about the putative role of such disruption on the occurrence of genetic alterations observed in MGUS and MM in the light of this hypothesis. The following paragraphs try to detail the possible cellular/tissue events that could lead to such MGUS/MM initiation.

### What is the cell of origin for MGUS and MM?

As previously mentioned, MGUS is now recognized as the obligatory step before MM development. Nevertheless, numerous polyclonal B cell expansions occur during the lifespan of an individual and only rare cases are stabilized in MGUS. Indeed, MGUS have to be distinguished from transient reactive plasmacytoses that spontaneously regress within 1 or 2 days. While becoming clinically detectable, MGUS are already stabilized forms of B cell expansion. This questions the nature of the events at the origin of this stabilization and implies that (1) some events occurring before, during or after the initial expansion phase make the expansion persists and create MGUS; (2) a “pre-MGUS” phase exists and constitutes the very early phase that has to be studied to decipher the molecular, cellular and/or environmental events at the origin of MM. Understanding the biology of these lesions is crucial to understand the earliest events in the evolution of these tumors (55).

The question of the cell of origin for MGUS and MM is a long-standing debate. MGUS is an almost non-proliferative disease while the transition to MM is characterized by proliferation of PC. Normal B memory cells generally contain the same genetic abnormalities than the proliferating PC suggesting that they appear in more undifferentiated cells. Nevertheless, two models of PC malignancy co-exist. The first considers that neoplastic PC acquire oncogenic deregulation to escape the normal proliferation control to clonally expand while the second considers that the disease is supported by a population of clonally related B-cells that act as tumor progenitors to maintain the malignant population (77).

Various experimental arguments support the first model (77), especially the fact that B-cell populations related to the malignant PC clone are not universally detected (78), while other works suggest that origins of MGUS and MM reside in the germinal center B-cells (79). Indeed, recent results showed that a rare subpopulation of early oncogene-positive memory-like B cells in lymph node, blood, and bone marrow is descended from the cell of origin in the germinal center (80). These cells would differentiate into premalignant PC in the bone marrow, propagate through peripheral blood, and give rise to a benign neoplasia, clinically known as MGUS. In any case, reconsidering what the nature of the first event might be in the origins of MGUS and MM could explain the initial imbalance between proliferation and quiescence of PC in the way that could reconcile both antagonist models.

### What is the real importance of genetic alterations?

As previously mentioned, there are two main types of primary cytogenetic abnormalities in MM: trisomies and translocations involving the IGH gene. The trisomic form of MM is characterized by an extra copy of one or more odd-numbered chromosomes (chromosomes 3, 5, 7, 9, 11, 15, 17). These mutually exclusive abnormalities allow cytogenetic and molecular classifications that have been extensively reviewed elsewhere (81, 82). Of note, cytogenetic abnormalities are not systematically detected, what is generally interpreted as due to insufficient PC for analysis or likely reflecting the fact that cells have a rare abnormality that is not targeted by the probes used for detection (81). Nevertheless, one cannot exclude that those cases are characterized by a non-genetic driving force that might also be present in the majority of the cases, but generally not considered in the dominant genetic perspective.

The search for common mutational events in MM has led to the conclusion that there is no unifying mutation and high genetic complexity (82, 83). Additional intriguing sequencing results showed that nearly all the genomic changes and mutations found in MM can be observed in MGUS, and that very few, if any, additional mutations were detected at disease progression when serial samples were sequenced from the same individuals (67, 84–86). This suggests that disease progression from MGUS to MM is mainly determined by the loss of active extrinsic micro-environmental constraints (such as immune surveillance or niche-derived signals) (87). As previously mentioned, disappearance of OB in endosteal niches is a main feature of the MGUS > MM transition and MM is specifically characterized by their ability to kill OB that constitute one, maybe the main, constraint in the niche.

This also means that the observed clinical stability of MGUS lesions may depend predominantly on tumor-extrinsic growth controls mainly produced by OB and that the process of “malignant transformation” may depend more on how the tumor cells modify the host-mediated growth control (55). Finally, given their importance in disease progression, these constraints and their disruption might also be involved in the very early steps of the disease for the appearance of “pre-MGUS” that are still a complete mystery.

### Tissue disruption as the initial event

Several other arguments can be given for this involvement of a disruption of the OB-mediated micro-environmental growth control over PC in the appearance of MGUS. Especially, MGUS is always associated to bone diseases characterized by mineralization loss and decrease of OB activity such as osteoporosis. Bone content in benign MGUS is already highly decreased and could be either a cause or a consequence of MGUS. Moreover, MGUS incidence increases with aging. This is generally explained by a decrease in T cell immunity, but aging is also associated with enhanced bone remodeling and OB activity decrease.

Concerning disease progression, tumoral growth is highly accelerated when MM cells are injected in castrated mice harboring bone hyper-resorption. The rare forms of MM that still have high OB activity are far less aggressive and evolutive than common forms. Among the two main subtypes of MM, hyperploid forms are the more aggressive and actually characterized by stronger bone defects. Finally, co-culture of MM cells and OB showed inhibitory effects of OB that is likely due to Decorins and other interaction proteins (88). Efficient anti-MM treatments are also known to modulate OB activity, especially Velcade (89). Altogether, these data argue for the crucial role of OB-PC interactions not only for MGUS > MM transition and MM progression, but also for the appearance of MGUS.

When polyclonal activation of B cells occurs, especially in presence of immunodeficiency, the pools of memory B cells and plasmablasts increase. Several possible cases can be considered here: (1) genetically abnormal B cells preexist and are amplified along with normal B cells; (2) genetically abnormal B cells are generated during the expansion phase; (3) no genetic abnormality is present in any B cell at the end of the activation.

In the first two cases, it is easily conceivable that the proliferation disorder has a genetic origin. Both genetically normal and abnormal B cells proliferate at the same maximal rate but the latter will reach the non-proliferating terminal state more slowly or will remain as slow-proliferating cells, thus producing MGUS. Nevertheless, pre-existing abnormal B cells do not produce MGUS unless polyclonal activation occurs. Moreover, aneuploid B cells are highly frequent and do not systematically lead to MGUS following polyclonal activation of B cells, suggesting again that non-genetic factors favor MGUS stabilization. Thus, it appears that genetic abnormalities are not sufficient for stabilizing MGUS. But is it at least necessary?

In the third case, if no genetic abnormality is present in any B cell at the end of polyclonal activation, spontaneous evolution toward the non-proliferating differentiated state should theoretically occur for all cells, at least in the genetic perspective. However, PC have to home in the endosteal niche and to interact in the BRC to reach this terminal state, especially by interacting with OB and OC. As polyclonal expansion produces a high number of PC that have to home in the niches, it is entirely possible that some of them could not be able to establish these interactions because of the saturation and the full occupancy of the niches. In this case, some cells would remain non-fully differentiated and this phenomenon could by itself maintain a pool of immature PC. This phenomenon could be highly amplified if biological, mechanical or toxical processes disrupt endosteal niches and the BRC. Reduced number, size or functionality of endosteal niches would produce residual PC growth because many of them would not be fully differentiated. This would constitute the “pre-MGUS” phase and be sufficient to produce MGUS. Genetic and epigenetic abnormalities could appear at this stage, later in these pre-malignant steps and helping stabilizing MGUS. Of course, if already present, genetic abnormalities would accentuate PC proliferation but this model makes appear that initiation of MGUS might be entirely from non-genetic and micro-environmental origin. Here bone disorders are conceived as cause and not consequence of MGUS and genetic abnormalities act as amplifier and stabilizer of the disease, but they are not the initial cause.

In the same perspective, disease progression from MGUS to MM would be linked to further loss of the micro-environmental constraints exerted by endosteal niches as previously suggested (87). As OB disappearance is a main feature of the MGUS > MM transition, pre-malignant PC probably acquire the ability to kill OB and to establish vicious pro-oncogenic feedback loops with OC thanks to their intrinsic instability generated by the absence of full differentiation. Finally, this evolution toward osteoclastic malignancy would further amplify differentiation and proliferation defects of PC, and favor enhanced genetic and epigenetic instability. Classical branched evolution and appearance of spatial genomic heterogeneity would then follow and give the complex genetic composition of MM (82, 83, 90). Depending on the primary genetic abnormality, the nature and the number of the secondary modifications will differ but make tumors phenotypically converge toward symptomatic MM.

### A integrated model conciliating molecular, cellular and tissue influences

Transient plasmocytoses and MGUS are characterized by a higher proportion of less mature plasmablasts, suggesting that impaired differentiation process could be at the origin of these phenomena. Similar dedifferentiation processes are frequently described to be at the origin of benign tumors in many tissues (30). MM cells are also characterized by activated Notch and Hedgehog pathways that revealed a degree of dedifferentiation. Moreover, the aggressiveness of MM cells is correlated to their degree of dedifferentiation with translocation 4–14 being the more aggressive and the less differentiated. Dedifferentiation is clearly correlated to aggressiveness in MM.

As described above, tissue disruption would be the main origin of differentiation defects and the common necessary step for MGUS to appear. In our model, disruption of bone—PC interactions favors dedifferentiation of PC, and inhibition of OB and activation of OC appear during the disease progression because bone prevents proliferation and dedifferentiation. It is probably why osteoblastic/sclerotic MM is less aggressive. These cells remain under the partial control of a bone that is at least partially normal. The case of solitary MM that is also far less aggressive, more differentiated and non-osteolytic could also reveal the importance of the interplay between aggressiveness, differentiation and bone-PC interactions, and the role of the latter in producing and/or maintaining the PC terminal differentiation state.

Cellular interactions seem to be the main force allowing stabilization of SGE and thus cell differentiation (see the Introduction). One can suppose that these interactions stabilize gene expression in PC, enabling a stable differentiated and a non-proliferative state. But as soon as these interactions are disrupted or not possible, such as the ones mediated by Decorins between OB and PC, SGE could drive cell proliferation and dedifferentiation of PC, creating MGUS. Thus, BRC and endosteal niche disruption could be the common factor that favors the appearance of MGUS by maintaining immature cells following the initial polyclonal activation of B cells, preexisting genetic abnormalities being present or not before polyclonal activation. Moreover, progression toward MM is linked to the destruction of endosteal niches. Pre-existing bone disorders and toxical action of molecules acting on BRC cells and their interactions with PC can participate in this destruction. But destabilized pre-malignant PC cells could also acquire this ability to destroy endosteal niche thanks to their increased genetic and phenotypic plasticity.

From this point, each phenomenon that favors proliferation of the pre-cancerous cells is selected. Especially, co-evolution of PC cells and their stromal cells make inhibition of OB and activation of OC appear. Phenotypic heterogeneity due to SGE appears among PC, and is then submitted to a selective process resulting in decreased bone formation and increased bone resorption. CD45 heterogeneity could result from this initial enhancement of SGE. We propose that CD45 heterogeneity originates from initial disruption of bone—PC interactions, and that a selective process against CD45 expression then takes place.

Moreover, as soon as those intrinsically unstable immature cells persist in the body, genetic abnormality would necessarily appear. Indeed, cells maintained in a partially undifferentiated state because of loss of micro-environmental control harbor highly SGE that could favor the appearance of genetic and epigenetic lesions (1, 14). Following BRC failure to differentiate PC, their intrinsic gene expression instability is likely to reduce efficiency of DNA repair and epigenetic maintenance, allowing appearance new cancerous phenotypes and tumor evolution.

Finally the different hypotheses concerning the cell of origin mentioned above can be conciliated here. If aneuploid B cells pre-exist or are generated during polyclonal activation, it is expected that they would clonally expand and appear to be the cell of origin, but only tissue disruption create the permissive environment allowing the persistence of immature PC. It is probably the most common case. On the contrary, if genetic abnormalities appear only after tissue disruption and failure of full differentiation of PC, it would appear that these cells escape the normal proliferation control and constitute the cells of origin. A mix of these two extreme cases is possible if tissue disruption acts together with genetic abnormalities present in some B cells to produce a population of pre-malignant PC combining subpopulations that have or not clonally related B-cells.

In spite of the apparent genetic initiation, it appears that only failure of micro-environmental control over PC allows development of non-fully differentiated cells and appearance of MGUS and MM. Bone disorders are the common factor in all pre-malignant and malignant steps of the disease and seem to be obligatory. The variable nature of the genetic abnormalities, their apparent absence in rare cases, their high frequency in healthy tissues all call into question their role in the disease initiation. They more probably act as stabilizer to allow progression in a context that has already created a permissive environmental context.

## Conclusion

We developed here a model based on the initial BRC and endosteal niche disruption to conciliate both genetic and environmental data in MM. The idea of very early micro-environmental disruption is probably more developed for many carcinomas but its causative role in the initiation stage had never been detailed in molecular and cellular terms. This hypothesis will need further explorations but the several correlations highlighted in this review between bone changes on one side, and MGUS appearance and MM progression on the other side, drive new avenues for efficient microenvironment-based treatments in MM.

Cancer cells probably remain intrinsically unstable and plastic because they do not normally interact with their native microenvironment. In this perspective, targeting driver genetic events is clearly not sufficient because this phenotypic instability and plasticity would allow them to counteract these treatments. On the contrary, searching for molecules that stabilize gene expression and thus help to restore full differentiation and quiescence appears to be a valid alternative. In our perspective, only molecules that interact with the cancerous cells and “mimic” their original microenvironment would really be able to stabilize them. A two-steps strategy could be applied (30, 91) that considers the need to 1) first re-express the genes coding for proteins allowing these interactions (they are often repressed because they prevent proliferation and dedifferentiation); 2) then stabilize this re-expression by bringing in their environment the molecules that can interact with them.

Epigenetic-based treatments are clearly adequate for the first step. The second step requires identification of key interaction proteins that would “recreate” a non-permissive micro-environment, or the stimulation of cells that enable these interactions. In our case, Decorins or “pseudo-Decorins” as soluble proteins, and stimulation of OB proliferation, are good candidates. Providing peptides that mimic interactions domains of key environmental proteins could substitute for normal OB. Most treatments targeting the microenvironment in MM try to inhibit OC activity. Our work shows that it seems more important to reform bone and stimulate OB, or at least to mimic normal interactions with bone cells, to stop MM.

## Author contributions

J-PC and RB formulated the hypotheses and wrote the manuscript.

### Conflict of interest statement

The authors declare that the research was conducted in the absence of any commercial or financial relationships that could be construed as a potential conflict of interest.
